# Effectiveness and Applications of a Metal-Coated HNT/Polylactic Acid Antimicrobial Filtration System

**DOI:** 10.3390/polym14081603

**Published:** 2022-04-14

**Authors:** Antwine W. McFarland, Anusha Elumalai, Christopher C. Miller, Ahmed Humayun, David K. Mills

**Affiliations:** 1 Molecular Science and Nanotechnology, Louisiana Tech University, Ruston, LA 71270, USA; antwinemcfarland@gmail.com (A.W.M.J.); anushae@wustl.edu (A.E.); chris_miller@meei.harvard.edu (C.C.M.); ah.humayun@gmail.com (A.H.); 2 Center for Biomedical Engineering and Rehabilitation Science, Louisiana Tech University, Ruston, LA 71270, USA; 3 School of Biological Sciences, Louisiana Tech University, Ruston, LA 71270, USA

**Keywords:** antimicrobial, antiviral, blow spinning, halloysite, metal nanoparticles, nanofibers, polymer

## Abstract

A broad-spectrum antimicrobial respiration apparatus designed to fight bacteria, viruses, fungi, and other biological agents is critical in halting the current pandemic’s trajectory and containing future outbreaks. We applied a simple and effective electrodeposition method for metal (copper, silver, and zinc) coating the surface of halloysite nanotubes (HNTs). These nanoparticles are known to possess potent antiviral and antimicrobial properties. Metal-coated HNTs (mHNTs) were then added to polylactic acid (PLA) and extruded to form an mHNT/PLA 3D composite printer filament. Our composite 3D printer filament was then used to fabricate an N95-style mask with an interchangeable/replaceable filter with surfaces designed to inactivate a virus and kill bacteria on contact, thus reducing deadly infections. The filter, made of a multilayered antimicrobial/mHNT blow spun polymer and fabric, is disposable, while the mask can be sanitized and reused. We used several in vitro means of assessing critical clinical features and assessed the bacterial growth inhibition against commonly encountered bacterial strains. These tests demonstrated the capability of our antimicrobial filament to fabricate N95 masks and filters that possessed antibacterial capabilities against both Gram-negative and Gram-positive bacteria.

## 1. Introduction

Infectious diseases are a leading cause of mortality worldwide, with viruses severely impacting healthcare and socio-economic development [1,2]. With the COVID-19 pandemic, we faced a global health crisis not seen since the influenza pandemic of 1918. The COVID-19 pandemic has caused severe disruptions to human communities and social services, massive unemployment, the liquidation of cash reserves, supply chain imbalances, and escalating economic turmoil that nations have witnessed across the globe. As a result, much of our social fabric, lifestyles, and educational institutions have changed. Recent and widespread vaccinations have brought some aspects of life back to normal; however, life will remain different for the foreseeable future. However, the emergence of the Delta and Omicron variants has shown how vulnerable we remain.

The recent onslaught of the COVID-19 pandemic, caused by a novel coronavirus, has affected every facet of human life. The human mouth acts as an incubator for certain viruses and is susceptible to viral entry [3]. The presence of the angiotensin converting enzyme-2 receptor (ACE2) expressed by epithelial cells of the mouth and throat cavity makes the virus binding challenging to resist [4,5]. Recent research has shown evolutionary advantages of the virus in binding to the host surface, a longer-lasting viability, and an increase in active receptor sites in humans [6,7]. 

The transmission of COVID-19 is thought to occur through respiratory droplets. Current Center for Disease Control and Preventions (CDC) guidelines recommend using N95 masks for health care providers managing the care of patients infected with SARS-CoV-2 or persons under investigation (PUI) for COVID-19 [1,2]. However, the global shortage of personal protective equipment (PPE) in the setting of the recent viral pandemic has created potentially dangerous conditions for frontline healthcare workers lacking appropriate protection for themselves and their patients [8]. In the spring of 2020, there existed a shortage of N95 masks, surgical masks, and face shields at hospitals for medical responders testing and treating COVID-19 patients. Medical responders must be protected from respiratory droplets originating from patients coughing and sneezing [9].

COVID-19 particles range from 60–140 nm, while N95 masks filter particles at 300 nm and only 95% of test particles. N99 respirators filter particles at 100 nm while not inactivating pathogen particles. Any smaller particles that are filtered by the N95 or N99 mask are done so by electrostatic forces. Individuals with chronic respiratory, cardiac, or other medical conditions may face difficulties wearing N95 masks for extended periods due to labored breathing. In addition, due to the high similarities of previous novel coronaviruses, it is believed that immunocompromised individuals, the elderly, and patients suffering from chronic or acute respiratory illness remain highly susceptible to infection and exhibit higher mortality rates than the general public [10,11]. The infectious nature of this virus remains a threat to all of society as all age groups have been impacted to some degree. Moreover, N95 respirators are not designed for children or individuals with facial hair. As a result of these inherent gaps in capability, a new mask must be developed for an increased protection against viral pathogens while considering other factors, such as comfort and mask-related health risks.

The immediate and enduring need for protective masks continues to mount each day for healthcare professionals worldwide. Due to the fact that the future timing of outbreaks is unknown, developing a mask that protects a broader scope of wearers in a clinical or domestic setting remains a high priority. All healthcare personnel, ordinary citizens, and our service members and their families should not have to resort to homemade apparatus that cannot prevent the spread of viral particles [12]. There are shortages of masks, current N95, and other makeshift garments that do not filter viral particles completely. Even though many individuals and groups are designing and manufacturing face shield frames for these responders, many designs are not implemented for high-density optimized additive manufacturing. As a result, they cannot be accessed locally [12,13]. There is a significant problem needing to be addressed immediately. Otherwise, we may witness wide-reaching impactful changes leading to substantial social upheaval [14].

N95 and surgical respirators are personal protective equipment to protect the wearer from airborne particles and liquid contaminants [15]. An N95 respirator is a respiratory protective device designed to achieve a very close facial fit and efficient filtration of airborne particles. Some N95 respirators are intended for use in a healthcare setting and are worn by healthcare personnel during procedures. The N95 mask is designed to protect both the patient and health care personnel from the transfer of microorganisms, body fluids, and particulate material, and to filter specific amounts of viruses or bacteria by reducing the amount but not actively killing viruses, bacteria, or fungi [16,17,18,19]. A recent publication noted that the co-infection with other respiratory pathogens was as high as 21% [20]. There is a common misconception that surgical masks provide adequate wearer protection from the inhalation of harmful microorganisms. Surgical masks protect a patient’s wounds from aerosol or mucosal droplets containing harmful microorganisms but provide little respiratory protection for the user [21].

Citizens increasingly wear FFP2 (filtering facepiece) in continental Europe, a medical-grade mask. It consists of a highly efficient, multi-layered fabric, including a melt-blow polypropylene filter designed to trap the smallest airborne particles [22]. FFP2 masks filter approximately 94% of all aerosols, including airborne viruses such as COVID-19. America’s N95 and China’s KN95 masks provide a similar level of protection. A concern with the long-term wearing of N95 and FFP masks is the potential adverse health effects. A recent study reported a reduction in circulating O2 concentrations, shortness of breath, and light-headedness/headaches among FFP mask wearers [22]. For the N95 mask, an increased facial skin temperature, more significant discomfort, and lower wearing adherence were observed [23]. These studies suggest the need for a new mask design that provides protection and is less impactful on the wearer’s health. The interaction of metal nanoparticles (mNPs) with microorganisms offers many advantages for therapeutic applications. mNPs offer unique physical properties that have associated benefits for drug delivery [23]. These are predominantly due to the particle size (affecting bioavailability and circulation time), large surface-area-to-volume ratio (enhanced solubility compared to larger particles), the tunable surface charge of the particle, and significant drug payloads that can be accommodated [24,25]. 

Silver and copper exert toxicity at inherently low concentrations that are non-toxic to mammalian cells [26,27]. When mNPs’ particle size is reduced, this amplifies the toxicity, even at low levels, and increases their effects on prokaryotes [25]. The combination of silver, copper, and zinc nanoparticles has been shown to exhibit synergistic antimicrobial activity, attributed to an increased prokaryotic cell permeability [27,28]. Furthermore, when mNPs are combined with antibiotics and doped into polymers, they have a similar but augmented antimicrobial effect [29,30].

In response to the COVID-19 pandemic, we designed an antimicrobial filtration system of medical-grade bioplastics and metal/ceramic nanoparticle composites [30]. The filter utilized in our design has a porosity of 50 nm due to the nature of the electrospun fibers that form a “spider-web-like” material that is breathable and long-lasting. Our first application of this filter system is a replacement for N95 masks. The filter unit can be disinfected via disinfectant cleaners, alcohol, or UV light exposure. Critical to our design concept was fabricating a fluid-resistant filter unit and providing the wearer with protection against large droplets, splashes, or sprays of bodily or other hazardous fluids. It also protects the patient from the wearer’s respiratory emissions and reduces exposure to particles, including small particle aerosols and large droplets (only non-oil aerosols). The filtration system was attached to our proprietary antimicrobial thermoplastic mask. 

## 2. Materials and Methods

### 2.1. Fabrication Design

The critical design feature in N95 respirators and surgical masks is the filtration unit. We have an efficient and effective design using 3D printing that can be scaled up for mass production via injection molding or industrial-scale 3D printers. Filtration units can be fabricated with any desired size or thickness, elasticity, or choice of thermoplastic. Polycaprolactone (PCL), polylactic acid (PLA), polypropylene (PP), and thermal plastic urethane (TPU) are possible polymers of choice. We can also use these to make composite filter systems. Our medical textiles are composed of thermoplastic and halloysite nanotubes (HNTs). HNTs are ceramic nanoparticles mined in Utah, inexpensive (USD 15/metric ton), and widely used as a drug carrier and nanofiller to improve a polymer’s material properties. We also have a patented method for metalizing the HNT surface with antimicrobial metals (copper, silver, zinc, and others). As 3D printing will assemble the masks in a layer-by-layer fashion, it affords a significant ability for customization with different metal-coated HNTs (mHNTs), antimicrobial-loaded HNTs, and antimicrobial-loaded mHNTs. The 3D-printed textiles also carry an electrostatic charge. 

### 2.2. Inner and Outer Fabric Layers

Many N95 masks utilize a triple-ply approach to manufacture their respirators. These three-ply materials are typically made of an outer layer of bonded fabric, a filtration layer comprising melt-blown material, and an inner layer made of more bonded fabric. A similar approach was taken for the manufacturing of our filters. Our filter for the masks also comprised three layers. The first and last layers comprised rayon/polyester wound gauze pads (Equate, Bentonville, AR, USA). The inner layer comprised blow spun PLA/mHNT. The outer (bonded) fabric can be easily impregnated with antimicrobials applied to the fabric or incorporated into the fabric’s structure. A multilayered approach to manufacturing the filters offers an improved ability to filter particles in both airflow directions (inhalation and exhalation).

### 2.3. Material Preparation and Filament Extrusion

#### 2.3.1. Metal HNT Preparation

A non-sacrificial standard two-electrode electrolysis setup consisted of two platinum-coated titanium mesh electrodes acting as a reversible cathode and anode. The electrodes were gently cleaned using silicon carbide abrasive papers and ultrasonicated in distilled water for 10 min to remove any surface contamination. The electrodes were held parallel at a 2-inch distance and connected to a DC power source (VWR Accupower 500 electrophoresis power supply).

An ultrasonicated colloidal solution of 700 mL of 5 mM (AgNO3, CuSO4, or ZnSO4, respectively) and 350 mg HNT were dispersed in the electrolysis vessel (1000 mL VWR borosilicate glass beaker) and stirred constantly using a magnetic stir bar to reduce electrophoretic buildup and precipitate formation at the working electrode. A temperature of 80 °C during the electrolysis process was maintained. The 20 V charge was applied in 5 min intervals, after which, the polarity was reversed. This process continued for a total of six 5 min cycles. The supernatant was decanted and washed with deionized water three times. The solution was centrifuged at 2000 rpm for 5 min with water to separate mHNTs from the unreacted NPs. The supernatant was removed, and 4 mL of ethanol was added to suspend the mHNTs. The mHNT and ethanol mixture was then dried at 37 °C. 

#### 2.3.2. HNT and mHNT PLA Filament Preparation

Five compositions were tested in this study: PLA, PLA+mHNTs/Zn, PLA+mHNTs/Cu, PLA+mHNTs/Ag, and PLA+mHNTs/(Ag, Cu, Zn). Filaments were extruded using a Noztek Pro Extruder (West Sussex, England) with a uniform diameter of 1.75 ± 0.05 mm. For the PLA group, PLA filaments were extruded at 175 °C. For PLA+mHNTs groups, to archive a uniform distribution of HNTs in PLA, 20 µL of PEG 200 was added into 20 g PLA and vortexed for 10 min, then 1 g of Zn, Cu, or Ag mHNTs were added and continually vortexed for another 10 min. For PLA+mHNTs/ (Ag, Cu, Zn) mixture, 0.33 g of each respective mHNT type were combined before adding them to the PEG-coated PLA. All mixtures of filaments were extruded at 181 °C. Before extrusion, all compositions were allowed to pre-dry for 4 hrs in an incubator at 60 °C, as per the manufacturer’s instructions, to yield a more uniform extrusion. 

### 2.4. Three-Dimensional Printing of Masks

Three-dimensional printing of the shows was conducted on an Ender 3 3D printer with the most general settings. The mask’s design was produced using the accessible computer-aided design (CAD) website TinkerCAD.com and converted to a stereolithography (.sol) file. Ultimaker Cura 4.5 was used to adjust design parameters and create the g-code. Each mask was printed at 210 °C with an infill ratio of 50%, and the print platform was heated to 50 °C to aid in bed adhesion. 

### 2.5. Three-Dimensional Printing of Testing Discs

Each filament was printed into test discs for bacterial studies using a Creality ENDER 3 (Shenzhen, China) 3D printer with similar settings. Due to the addition of the mHNTs, a slightly higher temperature was needed to have successful prints of the testing discs. Therefore, the PLA filament was printed at 205 °C, whereas the PLA/mHNT filament was printed at 210 °C. The test discs were cylindrical, with a height of 2 mm and a diameter of 6 mm, for an overall surface area of 94.25 mm. The infill ratio of the discs was 100% to make a solid disc with minimal porosity. 

### 2.6. Antibacterial Testing

Bacterial cultures were prepared to test the ability of the pre-extruded and 3D prints filament to facilitate the inhibition of bacterial growth. Antibacterial testing was performed on both GS-doped mHNTs and undoped mHNTs. Mueller–Hinton agar plates and Mueller–Hinton broth test tubes were used to provide testing mediums for the antibacterial capabilities of the doped mHNTs. Undoped pre-extruded non-doped mHNTs were only subjected to liquid medium antibacterial testing in Mueller–Hinton broth. 

#### 2.6.1. Muller–Hinton Broth

Liquid medium testing was facilitated using Mueller–Hinton broth. We used two nosocomial bacteria, *E. coli* and *S. aureus*, to model Gram-negative and Gram-positive bacterial sources. Testing was performed on mHNT-coated PLA beads and gentamicin-doped mHNT-coated PLA beads. Finally, it was performed on 3D-printed gentamicin-doped mHNTs PLA discs. Glass culture tubes were inoculated with 50 µL of *E. coli* or *S. aureus* after each respective mHNT, or doped mHNT was added to the broth of each individual tube. Controls of uninoculated Mueller–Hinton broth and inoculated broth were used. The cultures and controls were incubated at 37 °C for 48 h. Readings were taken for the mHNTs at the 0 and 24 h mark. In addition, readings were taken for the GS-doped mHNTs beads and discs at the 0, 24, and 48 h mark. Each test was carried out in triplicate, and the results were averaged.

#### 2.6.2. Muller–Hinton Agar

Mueller–Hinton agar plates were prepared to the manufacturer’s specifications. We used *E. coli* and *S. aureus* as Gram-negative and Gram-positive bacterial sources. Testing was performed on 3D-printed gentamicin-doped mHNTs PLA discs. Each respective filament blend was printed into the small cylindrical disc described above. Each polymer was plated added to the bacteria-covered agar plates before a 24 h incubation at 37 °C. Zone of inhibition measurements was taken using a digital caliper. Each test was carried out in triplicate, and the zone of inhibition diameters was averaged. 

### 2.7. Solution Blow Spinning of mHNT Fibers

A solution of 15 mL dichloromethane (DCM), 500 mg PLA, and 100 mg ZnHNTs were combined and ultrasonicated for 20 min. The solution was then left for 24 h at room temperature. A Zen gravity feed airbrush kit was used with the air pump set to maintain a constant pressure of 40–60 psi. A square (4 in × 4 in) of sterile gauze was attached to the spraying platform. A total of 4 mL of the DCM/PLA/mHNT solution was evenly airbrushed onto the surface of the gauze. The gauze was allowed to dry overnight fully. Two squares were then placed together with the airbrushed sides facing each other to make a single filter.

### 2.8. Statistical Analysis

Statistical analysis was performed using Microsoft Excel Analysis ToolPak plugin and Origin 9.6. All experiments were carried out in triplicate, and a one-way analysis of variance 34 (ANOVA) with *p* < 0.05 as the significance level was utilized for statistical analysis. Statistically significant data were reported (*p* < 0.05), and all of the results were reported as mean ± standard deviation (*p* < 0.05, *n* = 3) unless otherwise specified.

## 3. Results

### 3.1. Liquid Growth Inhibition Studies

**Figure 1:**
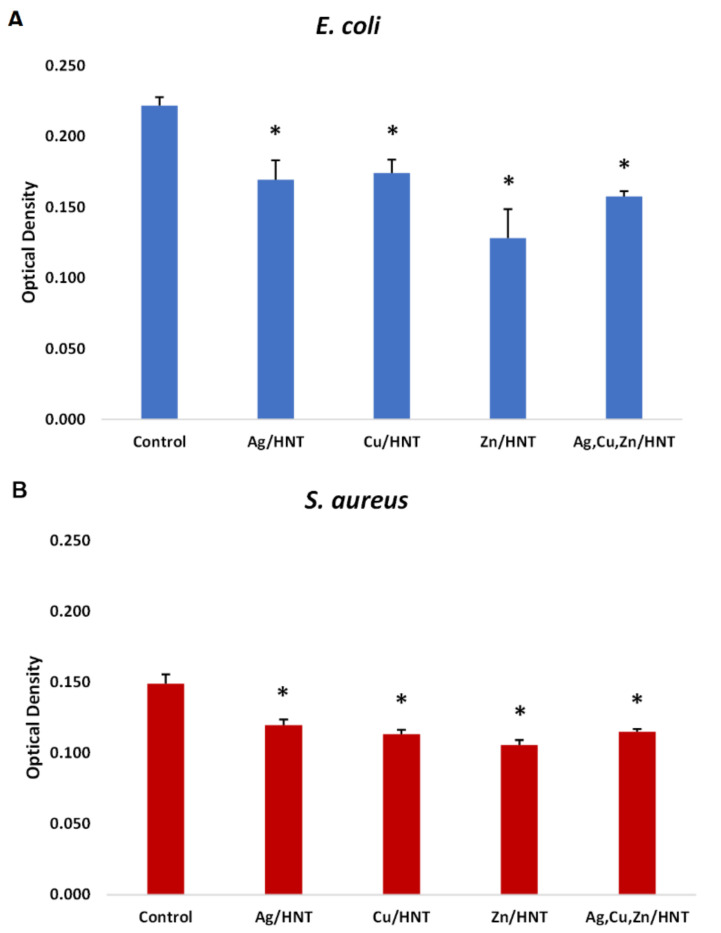
PLA beads coated with mHNTs in Mueller-Hinton broth for 48 h with *E. coli* (**A**) or *S. aureus* (**B**). Optical density was taken at 630 nm wavelength. Error bars represent ± standard deviation. Asterisk (*) indicates a significant difference (*p* < 0.05) to control (*n* = 3).

**Figure 2:**
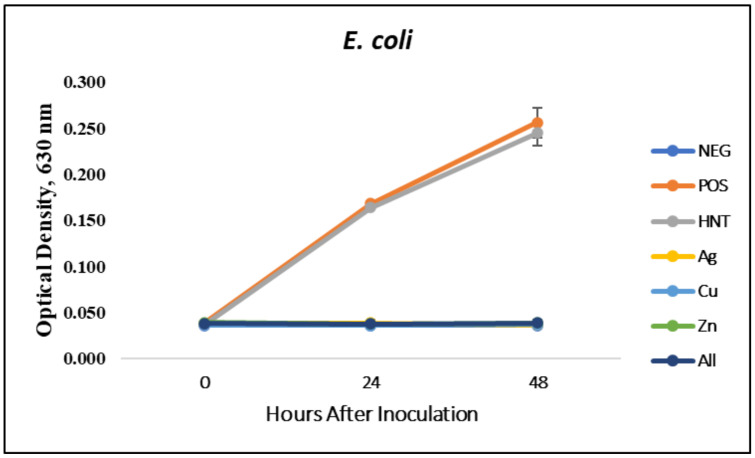
PLA beads coated with GS-doped mHNTs in Mueller–Hinton broth for 48 h with *E. coli*. Optical density readings were taken at 0, 24, and 48 h. Optical density was taken at 630 nm wavelength. Error bars represent ± standard deviation (*n* = 3).

**Figure 3:**
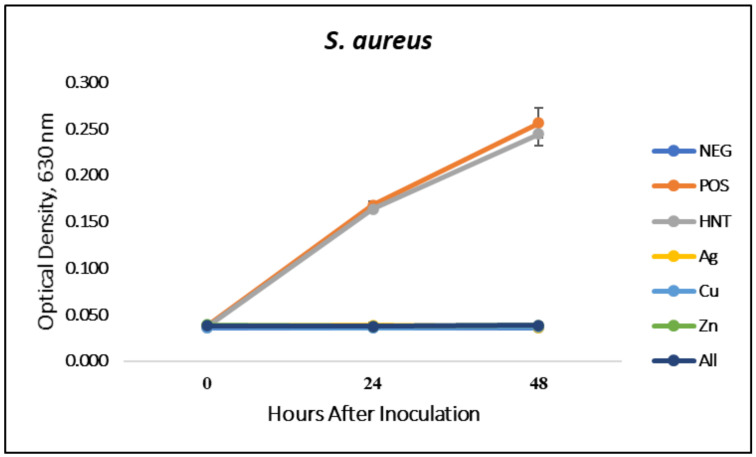
PLA beads coated with GS-doped mHNTs in Mueller–Hinton broth for 48 h with *S. aureus*. Optical density readings were taken at 0, 24, and 48 h. Optical density was taken at 630 nm wavelength. Error bars represent ± standard deviation (*n* = 3).

**Figure 4:**
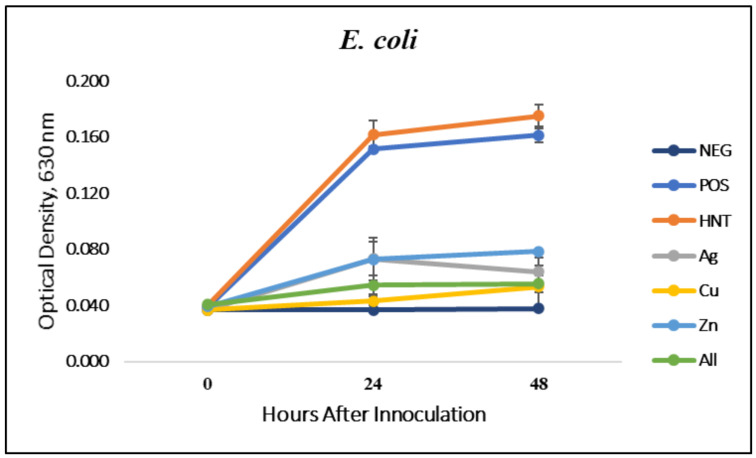
Three-dimensional-printed PLA with GS-doped mHNTs in Mueller–Hinton broth for 48 h with *E. coli*. Optical density readings were taken at 0, 24, and 48 h post-inoculation. Optical density was taken at 630 nm wavelength. Error bars represent ± standard deviation (*n* = 3).

**Figure 5:**
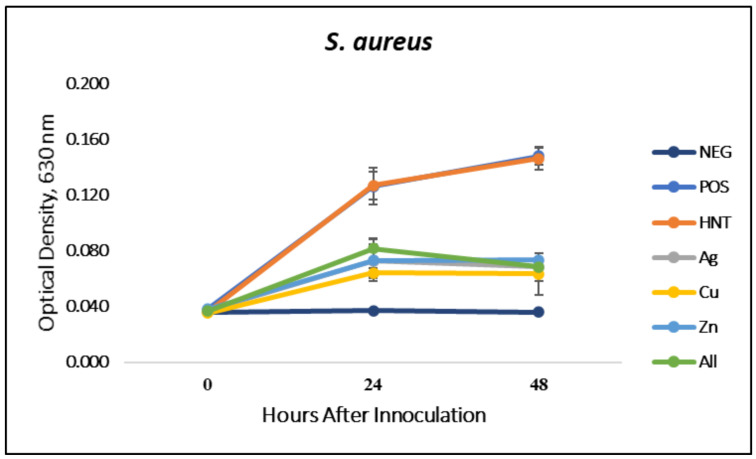
Three-dimensional-printed PLA with GS-doped mHNTs in Mueller–Hinton broth for 48 h with *S. aureus*. Optical density readings were taken at 0, 24, and 48 h post-inoculation. Optical density was taken at 630 nm wavelength. Error bars represent ± standard deviation (*n* = 3).

The absorbance values for each Mueller–Hinton broth culture were measured at 630 nm. The results for the mHNT-coated PLA beads are shown in Figure 1. The results for the 24 h and 48 h cultures that contained GS-doped mHNT-coated PLA beads are shown in Figure 2 and Figure 3. Figure 4 and Figure 5 show the results of the 3D-printed GS-doped mHNTs. The optical density is directly correlated to the turbidity of the broth. An increased turbidity is evidence of higher bacterial growth. Accordingly, the control broth for each set of cultures yielded the highest optical densities. This outcome was observed for *E. coli* and *S. aureus* cultures at the 24 h and 48 h readings. 

In the studies carried out with GS-doped mHNT-covered PLA beads and 3D-printed GS-doped mHNT-embedded PLA discs, the cultures were also carried out with HNT-coated PLA beads and 3D-printed PLA embedded with HNT to see the effects of plain HNTs on bacterial growth. The resulting studies showed little to no growth inhibition of either *E. coli* or *S. aureus*. The GS-doped mHNT-coated beads showed little to no bacterial growth after an incubation period of 24 h and 48 h.

The bacterial studies carried out with 3D-printed GS-doped mHNT-embedded PLA discs (Figure 4 and Figure 5) show a higher optical density than the GS-doped mHNT-covered PLA beads, as seen in Figure 2 and Figure 3. 

### 3.2. Agar Plate Growth Inhibition Studies

**Figure 6:**
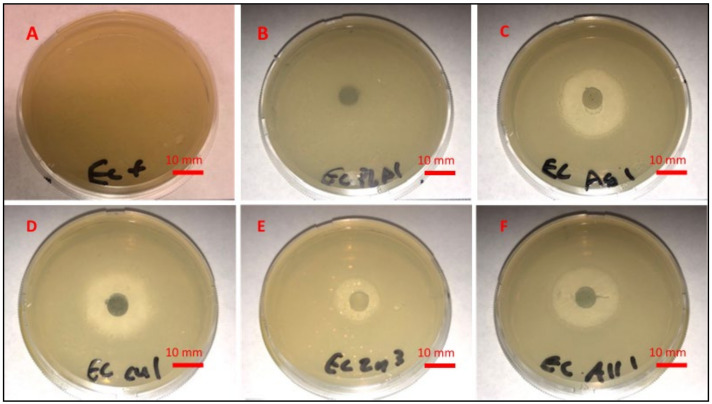
Mueller–Hinton agar plates, plated with *E. coli*, and (**A**) no sample, (**B**) PLA, (**C**) 3D-printed GS/Ag/HNT disc, (**D**) 3D-printed GS/Cu/HNT disc, (**E**) 3D-printed GS/Zn/HNT disc, and (**F**) 3D-printed GS/Ag, Cu, Zn/HNT disc.

**Figure 7:**
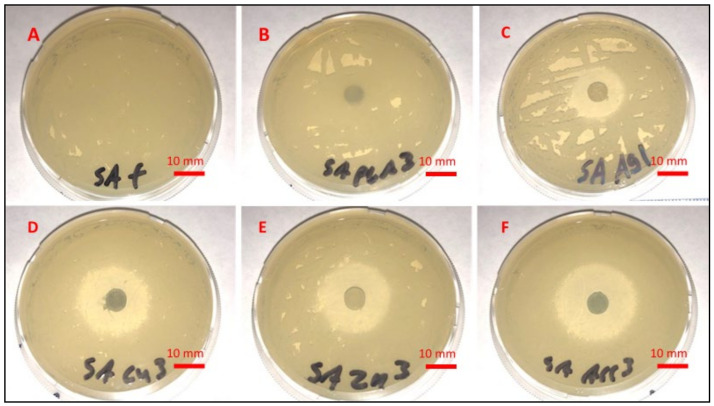
Mueller–Hinton agar plates, plated with *S. aureus*, and (**A**) nothing, (**B**) PLA, (**C**) 3D-printed GS/Ag/HNT disc, (**D**) 3D-printed GS/Cu/HNT disc, (**E**) 3D-printed GS/Zn/HNT disc, and (**F**) 3D-printed GS/Ag, Cu, Zn/HNT disc.

**Figure 8:**
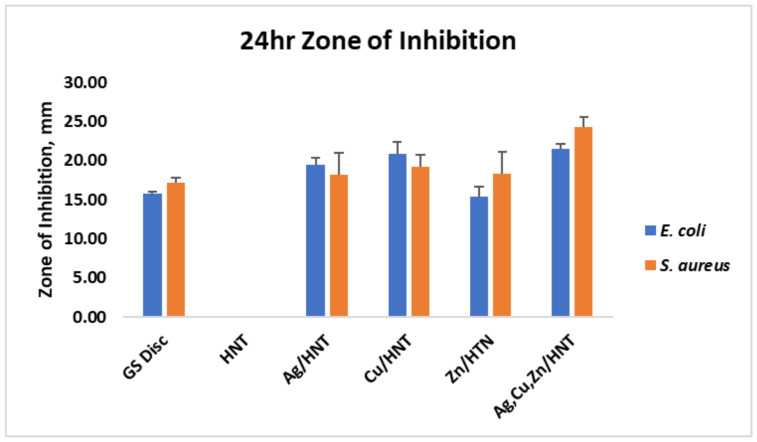
Zone of inhibition diameter average for each respective 3D-printed disc after 24 h. These were GS-loaded mHNTs. Error bars represent ± standard deviation.

Figure 6 and Figure 7 display the zone of inhibition resulting from different combinations of 3D-printed PLA discs. A positive control lawn of bacteria was used for both *E. coli* and *S. aureus* to demonstrate the average bacterial growth on the Mueller–Hinton agar plates. A PLA embedded with plain HNTs was also plated to determine if there were any antibacterial properties of plain HNTs. Figure 8 shows the average zone of inhibition for each respective 3D-printed polymer disc. 

### 3.3. Fabrication of the N95 Mask and Filter Assembly

**Figure 9:**
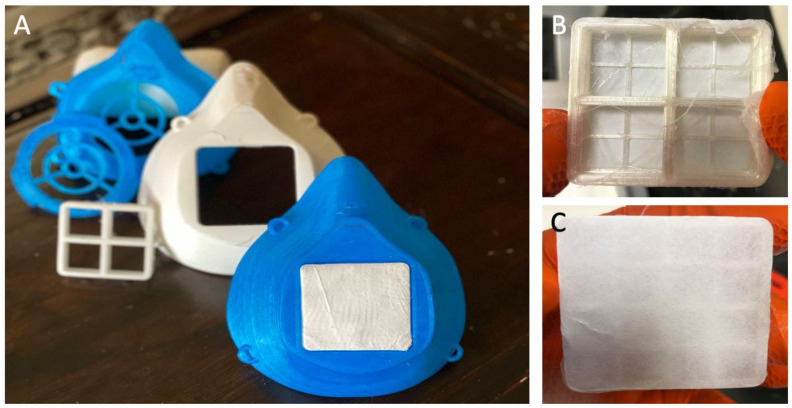
(**A**) Example of N95 masks and filter frameworks 3D printed with a customized Cu/HNT printer filament. (**B**,**C**) Zn/HNT filter blow spun on a 3D-printed Cu/HNT filter frame.

**Figure 10:**
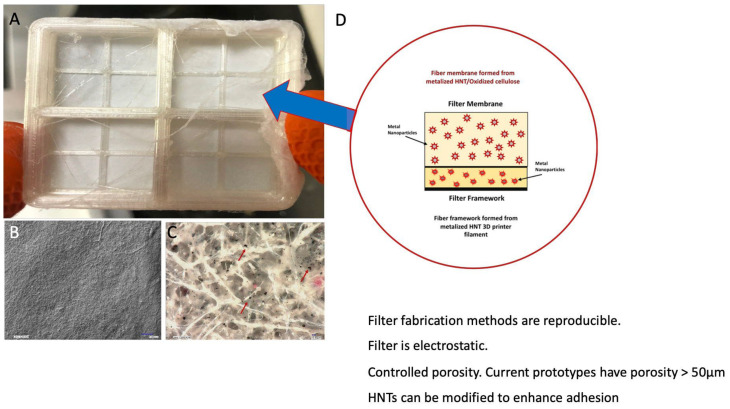
(**A**) Filter framework and blow spun ZnHNT fibers, (**B**) digital image of filter surface, (**C**) high-power view of ZnHNT fibers (ZnHNTs indicated by red arrow) and (**D**) filter composition.

Our antimicrobial/antiviral filtration system is made of medical-grade bioplastics. The core technology is metalized 3D printer filament and blow spun fibers (Figure 9). In Figure 10, we see more detailed images and a diagram of the composition of the zinc/HNT filter material. We used a method to coat antimicrobial/viral metal nanoparticles using a “green method” that does not generate toxic waste and has a low environmental impact. 

Our method is a cost-efficient and straightforward method for metalizing halloysite nanotubes (HNTs) with environmentally benign copper, silver, and zinc nanoparticles (mHNTs). Metal nanoparticles will deactivate a virus, reduce or eliminate bacterial adhesion, prevent bacterial growth, and reduce deadly infections. The mask is comfortable, flexible, and non-rigid. Testing by immersion in warm water showed that it possesses a malleable nature, providing a form-fitting design.

Filtration units can be fabricated with any desired size or thickness, elasticity, or choice of thermoplastic. The filtration systems are also autoclavable and compatible with disinfectant cleaners and UV systems. 

## 4. Discussion

Eliminating the devastating societal impact of viral pandemics and bacterial and fungal infections will be driven by innovations and discoveries resulting from the convergence of medicine with cell and molecular biology, engineering, chemistry, and material science. These collaborative efforts will lead to a greater understanding of disease etiology and technological breakthroughs in diagnosing, treating, and preventing infectious diseases [31,32].

3D printing has demonstrated its usefulness in providing biomedical solutions to many clinical problems [33,34,35]. Three-dimensional printing techniques are mainstream and commonly used in the aerospace, construction, defense manufacturing, medical devices, and pharmaceutic industries [34,36,37]. As 3D printing has advanced, novel materials (bioplastics, ceramics, metals, nitinol, high-temperature nylon, and superalloys) have significantly increased unique properties [38,39]. For example, creating new custom-made filaments with antimicrobial applications offers protection against infectious outbreaks and viral pandemics, especially COVID-19 [15,39,40,41,42,43,44].

In this study, the antibacterial capabilities of mHNTs and GS-doped mHNTs were tested using Gram-positive and Gram-negative bacteria. All iterations of mHNTs impacted the rate of bacterial growth. We were able to show that any possible antibacterial effects of the mHNT/PLA were not due to the polymer or HNT, which satisfies our null hypothesis. This was evident due to the poor antibacterial effects of the uncoated HNTs when exposed to bacteria. However, similar bacterial growth restrictions have been seen in various metal nanoparticle experiments [45,46,47,48]. While the scientific community does not entirely agree on all of the mechanisms of the metals, we do have some insight into a few mechanisms that affect the cytotoxic capability of bacteria and other microorganisms. The most accepted mechanism for zinc and copper nanoparticle antibacterial effectiveness is the formation of reactive oxygen species and, upon contact with the walls, the destruction of the integrity of the cell [45,46]. These reactive oxygen species compromise the cell’s osmotic stability, causing cell death [45,46]. Alternatively, gold nanoparticles are not effective due to reactive oxygen. Their anti-bacterial effect is attributed to a decreased ribosomal binding of tRNA and ATPase inhibition in Gram-negative bacteria [47]. Ortiz-Benitez et al. examined the antibacterial effects of gold nanoparticles on Gram-positive *Streptococcus pneumoniae* [48]. They revealed that the bactericidal effect, which eventually led to cell lysis, was due to unrepairable pores in the cell membrane caused by the accumulation of recruited carbohydrates, lipids, and proteins [48]. 

Vacuum loading mHNTs with GS enhanced their ability to inhibit bacterial growth. Figure 2, Figure 3, Figure 4 and Figure 5 show that the non-3D-printed GS-loaded mHNT-coated PLA beads were more bactericidal than the 3D-printed GS-loaded mHNT discs. We speculate that, since the entire load of GS-loaded mHNTs was on the surface of the beads, more of the nanoparticles and gentamicin were available to deter bacterial growth. In addition, the 3D-printed discs were made with extruded filament containing the GS-loaded mHNTs, whereas the GS-loaded mHNTs were dispersed throughout the filament and, accordingly, the 3D-printed discs.

While the coated beads performed better than the 3D-printed discs, they lost their ability to be an effective bacterial deterrent over time. The nature of the 3D-printed discs is that they break down more rapidly over time, as similarly described in Hakkarainen et al. [49]. This effect should see the loss of empty mHNTs exposed at the surface, where they are released, resulting in new GS-doped HNTs to the surface. Thus, it should retain its antimicrobial properties for a more extended period. As similarly described in Tappa et al. in 2018, the principle of a renewed antimicrobial outer surface offers many benefits for manufacturing products such as masks, light switches, and toilet handles [34]. 

The introduction of antibiotics in the 20th century provided short-lived relief from almost all bacterial infections; however, with time, bacteria evolved, and cases of antibiotic-resistant strains are on the rise. In addition, the antibiotics currently in use also suffer from systemic toxicity and a short half-life, and have led to the emergence of multidrug-resistant (MDR) bacteria. Therefore, measures to overcome recurrent healthcare-acquired infections and increased resistant strains are a critical research priority [43,49]. 

Medical facilities, such as hospitals or clinics, could see the most benefit from using 3D-printed antimicrobial materials in the fight against the spread of MRSA bacteria. Surfaces are usually sanitized in medical facilities, but there are time gaps before these surfaces are cleaned again [44]. Surfaces such as doorknobs, water fountains, and water faucet handles are often touched, with little thought about the possible transfer of infectious agents [41]. Our technology can significantly reduce or inhibit bacterial adherence and colonization, and prevent biofilm formation. An innate ability to self-sanitize would offer considerably better protection to all who come into contact. 

Two large-scale manufacturing methods used to produce antimicrobial devices and materials in the quantities needed are industrial 3D printing or injection molding [50]. Each method of manufacturing has its advantages and disadvantages. The benefits of industrial 3D printing are the rapid ability to change the design of the object being printed. The obstacles faced with industrial 3D printing are the slow turnover and increased porosity [15]. Injection molding has faster throughput, but it is generally harder to adjust the design.

The GS-doped mHNTs and their antimicrobial capabilities can be used to manufacture a range of biomedical and industrial products other than the face masks and filters tested in this study. For example, intubation tubes and catheters can be manufactured with mHNTs to reduce infections in patients caused by bacteria [51]. Additionally, transparent materials such as eye guards made using mHNTs embedded in polycarbonate or poly- (methyl methacrylate) [52] and glass [53] have been fabricated. The polymer clarity was not compromised by incorporating the mHNTs and showed an improvement in strength and antimicrobial capability.

## 5. Conclusions

3D printing has emerged as a viable option to manufacture face masks. We have demonstrated the capability of our antimicrobial filament to be used to manufacture an act that has antibacterial capabilities. GS-loaded mHNTs were shown to successfully kill or retard the growth rate of both Gram-negative and Gram-positive bacteria on surfaces and in liquid mediums. The Mueller–Hinton broth optical density and Mueller–Hinton agar plate zone of inhibition results support this conclusion. While the mHNTs showed an ability to hinder the growth of bacteria, the GS-doped mHNTs offered a much greater ability to kill or inhibit bacterial growth. Due to the filament manufacturing and the 3D processes, careful consideration was taken when choosing the antimicrobial agent used as a dopant. Future studies will explore alternative doping materials that can withstand the necessary heat to manufacture this product. We hope that the technology pioneered in these studies will be employed to manufacture 3D printer filament that can be made into customized surfaces that remain bacteria-free without excessive sanitation and comfortable, sanitizable antimicrobial face coverings. As staff and workforce efforts to sanitize surfaces often see ebbs and flows in the future, harmful microbes are inevitable, and measures to safeguard highly touched surfaces or filter them from the air are crucial for the health and safety of individuals.

## Data Availability

All data have been included in the manuscript.
